# Cytochalasin B Alleviates LPS-Induced Mastitis and Modulates the Expression of PI3K/N-WASP/ARP2/3-Related Molecules

**DOI:** 10.3390/biom16101467

**Published:** 2026-10-09

**Authors:** Weitao Dong, An Wang, Yaru Li, Xingcai Qi, Wenkai Bai, Kejiang Liu, Quanwei Zhang, Xingxu Zhao, Yong Zhang

**Affiliations:** 1 College of Veterinary Medicine, Gansu Agricultural University, Lanzhou 730070, China; 2 Gansu Key Laboratory of Animal Generational Physiology and Reproductive Regulation, Lanzhou 730070, China; 3 Lanzhou Modern Vacational College, Lanzhou 730030, China; 4 College of Life Sciences and Biotechnology, Gansu Agricultural University, Lanzhou 730030, China

**Keywords:** cytochalasin B, Mastitis, PI3K/N-WASP/ARP2/3, cytoskeletal regulation, inflammation

## Abstract

Cytochalasin B (CB) inhibits actin polymerization and modulates cytoskeletal dynamics, but its role in mastitis remains unclear. This study investigated the protective effects of CB against mastitis and its association with PI3K/N-WASP/ARP2/3-related molecular alterations. Transcriptomic analysis of mammary tissues from healthy cows and cows with subclinical or clinical mastitis revealed extensive transcriptional remodeling during mastitis progression. Validation showed enhanced inflammatory responses accompanied by increased expression of PI3K, N-WASP, CLTC, ARP2, and ARP3 in mastitic tissues. In LPS-stimulated bovine mammary epithelial cells (MAC-T), CB reduced pro-inflammatory cytokine expression, increased IL-10 expression, and attenuated the abnormal elevation of PI3K/N-WASP/ARP2/3-related molecules. Consistently, in an LPS-induced mouse mastitis model, CB alleviated mammary tissue injury and inflammatory cell infiltration, improved inflammatory cytokine profiles, and reduced the expression of PI3K, N-WASP, CLTC, ARP2, and ARP3. Collectively, CB attenuates LPS-induced mammary inflammation and tissue injury, and these effects are associated with alterations in PI3K/N-WASP/ARP2/3-related cytoskeletal molecules. These findings provide new evidence linking cytoskeleton-related molecular alterations to mastitis inflammation and the anti-inflammatory effects of CB.

## 1. Introduction

Bovine mastitis is one of the most prevalent and economically important diseases in dairy production. Its occurrence not only reduces milk yield and quality but also increases treatment costs, prolongs reproductive intervals, and raises the risk of premature culling [1,2]. Mastitis is commonly triggered by bacterial infections and their virulence factors. Among these, lipopolysaccharide (LPS), a major component of the outer membrane of Gram-negative bacteria, can activate innate immune responses in mammary epithelial and immune cells, promote the release of inflammatory cytokines, and disrupt mammary tissue architecture and barrier homeostasis. Although antibiotics remain an important treatment for bacterial mastitis, the increasing prevalence of antimicrobial-resistant strains, concerns regarding drug residues, and food safety issues have prompted growing interest in novel intervention strategies that modulate host inflammatory responses [3].

Mammary epithelial cells are not only responsible for milk synthesis and secretion but also constitute an important immune barrier against pathogen invasion [4]. Stimulation of mammary epithelial cells by pathogen-associated molecular patterns can induce innate immune responses and a series of cellular alterations, including inflammatory signaling, changes in epithelial junctions, and membrane-associated processes [4,5]. The actin cytoskeleton is not merely a structural framework that maintains cell morphology; it also plays essential roles in pathogen sensing, cell migration, phagocytosis, endocytosis, and vesicular trafficking [6,7]. Therefore, aberrant actin polymerization and cytoskeletal reorganization may represent important regulatory events contributing to persistent inflammation and tissue injury during mastitis.

Phosphoinositide 3-kinase (PI3K) is an important signaling molecule involved in the regulation of cell survival, metabolism, proliferation, and inflammatory responses and can influence multiple cytoskeletal regulatory proteins through phosphoinositide-dependent mechanisms [8,9,10,11]. Neural Wiskott–Aldrich syndrome protein (N-WASP) is an actin nucleation-promoting factor that regulates the activity of the actin-related protein 2/3 (ARP2/3) complex and promotes the formation of branched actin networks [12,13]. N-WASP/ARP2/3-mediated cytoskeletal remodeling plays critical roles in cell migration, pathogen internalization, immune synapse formation, and inflammatory responses [14,15,16,17]. In addition, clathrin heavy chain (CLTC), a major component of clathrin-mediated endocytosis, is closely associated with membrane receptor trafficking and cortical actin dynamics. Accordingly, aberrant expression or function of PI3K, N-WASP, ARP2/3, and CLTC may contribute to the crosstalk between cytoskeleton-related molecular alterations and inflammatory signaling during mastitis. However, changes in these cytoskeleton-related molecules and their biological significance in bovine mastitis have not yet been systematically investigated.

Cytochalasin B (CB) is a membrane-permeable fungal metabolite that binds to the barbed ends of actin filaments, thereby interfering with actin polymerization and cytoskeletal dynamics [18,19]. Previous studies have shown that CB can affect cell migration, endocytosis, oxidative stress, and inflammatory responses. In vascular smooth muscle cells exposed to high glucose, CB alters F-actin organization and reduces NADPH oxidase-associated oxidative stress [20]. During oocyte cryopreservation, CB pretreatment can alleviate cytoskeletal abnormalities and improve developmental competence [21,22]. In addition, previous studies have shown that CB can attenuate LPS-induced mammary inflammation, potentially through ARP2/3-dependent cytoskeletal alterations and inflammasome regulation [23,24]. However, whether CB also affects PI3K-, N-WASP-, and ARP2/3-related molecules, and how these changes are associated with the alleviation of mammary inflammation, remain incompletely understood.

Based on these considerations, the present study first examined the expression patterns of inflammatory cytokines and PI3K/N-WASP/ARP2/3-related molecules in mammary tissues from healthy cows and cows with subclinical or clinical mastitis. LPS-induced bovine mammary epithelial cell and mouse mastitis models were then used to evaluate the effects of CB on inflammatory responses, histopathological injury, and the expression of PI3K, N-WASP, CLTC, ARP2, and ARP3. This study aimed to clarify the anti-inflammatory effects of CB in experimental mastitis and to explore their association with alterations in PI3K/N-WASP/ARP2/3-related cytoskeletal molecules, thereby providing experimental evidence for further understanding the relationship between cytoskeletal regulation and mastitis-associated inflammation.

## 2. Materials and Methods

### 2.1. Collection and Processing of Bovine Mammary Tissue Samples

Twelve Holstein cows from a commercial dairy farm in Wuzhong, Ningxia, China, were assigned to healthy control (Control), subclinical mastitis (SCM), and clinical mastitis (CM) groups (*n* = 4 per group). Cows were comparable in age, parity, lactation stage, and body condition and were classified based on clinical examination, milk appearance, somatic cell count (SCC), and Lanzhou Mastitis Test (LMT) results. Cows with other systemic or reproductive diseases or recent treatment with antibiotics or anti-inflammatory drugs were excluded. Control cows showed no clinical abnormalities, with SCCs of 7 × 10^4^–1 × 10^5^ cells/mL and negative LMT results. SCM cows showed no overt clinical signs but had SCCs of 2 × 10^5^–5 × 10^5^ cells/mL and positive LMT results (+/++), whereas CM cows exhibited typical clinical signs and abnormal milk, with SCCs of 1.3 × 10^6^–1.5 × 10^6^ cells/mL and strongly positive LMT results (+++). One qualifying mammary quarter per cow was selected, and each cow was considered one biological replicate.

Following routine commercial slaughter, mammary parenchymal tissue was aseptically collected from the predefined quarter. Samples were divided into two portions: one was fixed in 4% paraformaldehyde for 24–48 h for histological analyses, and the other was snap-frozen in liquid nitrogen and stored at −80 °C for RNA and protein extraction. The sampling protocol was approved by the Animal Ethics Committee of Gansu Agricultural University (GSAU-Eth-LST-2021-003).

### 2.2. Animal Husbandry and Experimental Treatments

Eight-week-old SPF Kunming mice (50 females and 25 males) were obtained from the Chinese Academy of Agricultural Sciences and maintained under standard conditions (22 ± 2 °C, 50–60% humidity, 12 h light/dark cycle) with free access to food and water. After 7 days of acclimation, females and males were mated at a 2:1 ratio. Among the eligible lactating dams, 18 mice were randomly selected using a computer-generated random-number sequence. These mice were then randomly assigned to the Control, LPS, and LPS + CB groups (*n* = 6 per group). Under isoflurane anesthesia, mice in the Control group received 50 μL sterile saline into the fourth mammary gland, whereas the LPS and LPS + CB groups received 50 μL LPS (200 μg/mL; *Escherichia coli* O111:B4, L4391, Sigma-Aldrich, St. Louis, MO, USA). Pups were temporarily separated from the dams during anesthesia and intramammary administration and were returned after the dams had recovered from anesthesia. At 24 h after LPS administration, mice in the LPS + CB group received cytochalasin B (HY-16928, MCE, South Brunswick, NJ, USA) at 4 mg/kg by intraperitoneal injection. CB was initially dissolved in DMSO (5 mg in 70 μL) and subsequently diluted with PBS to the required concentration. The final concentration of DMSO in the injection solution was approximately 2% (*v*/*v*). Twenty-four hours later, mice were euthanized with an overdose of sodium pentobarbital (150 mg/kg), and mammary tissues were collected for molecular and histological analyses. The animal protocol was approved by the Animal Ethics Committee of Gansu Agricultural University (GSAU-Eth-LST-2021-003).

### 2.3. Transcriptome Sequencing and Bioinformatics Analysis

RNA sequencing was performed by Gene Denovo Biotechnology Co., Ltd. (Guangzhou, China) using mammary tissues from Control, SCM, and CM cows (*n* = 4 per group). Total RNA was extracted using TRIzol and assessed for concentration, purity, and integrity using a NanoDrop spectrophotometer (Thermo Fisher Scientific, Waltham, MA, USA) and Agilent 2100 Bioanalyzer (Agilent Technologies, Santa Clara, CA, USA). Qualified RNA was used for library construction and high-throughput sequencing. Approximately 74.1–90.5 million clean reads were obtained per sample, and the total mapping rates ranged from 91.12% to 94.65%. Clean reads were aligned to the *Bos taurus* reference genome using HISAT2. PCA and Pearson correlation analyses were performed to evaluate sample-level variability and reproducibility. Differential expression analysis was performed using DESeq2 with read counts as input. Read counts were normalized, followed by model-based hypothesis testing and multiple-testing correction. Genes with |log2(fold change)| ≥ 1 and FDR < 0.05 were considered differentially expressed genes (DEGs). Gene Ontology (GO) and Kyoto Encyclopedia of Genes and Genomes (KEGG) enrichment analyses were subsequently performed to characterize the biological functions and pathways associated with the DEGs.

### 2.4. Cell Culture, Treatments, and Cell Viability Assay

The bovine mammary epithelial cell line MAC-T was obtained from the Chinese Academy of Agricultural Sciences and cultured in DMEM/F12 supplemented with 10% fetal bovine serum at 37 °C in a humidified atmosphere containing 5% CO_2_. Cells at 60–70% confluence were assigned to Control, LPS, and LPS + CB groups. The LPS group was continuously exposed to 50 μg/mL LPS for 48 h. For the LPS + CB group, cells were initially exposed to 50 μg/mL LPS for 24 h, after which 500 ng/mL CB was added directly to the existing culture medium without removing LPS or replacing the medium, and the cells were incubated for an additional 24 h. For the additional control experiment, a CB-alone group was included, in which MAC-T cells were treated with 500 ng/mL CB for 24 h. CB was initially dissolved in DMSO (5 mg in 70 μL) and subsequently diluted with PBS. The final DMSO concentration in the MAC-T cell culture medium was approximately 0.0007% (*v*/*v*). Cell viability was assessed using a CCK-8 assay. MAC-T cells were treated with CB at 0, 0.5, 5, 50, or 500 ng/mL, or 5 or 50 μg/mL for 24 h. CCK-8 reagent was then added, and absorbance was measured at 450 nm after 1 h of incubation. The experiment was independently repeated three times, with three technical replicate wells for each treatment in each experiment.

### 2.5. RNA Extraction and Quantitative Real-Time PCR

Total RNA was extracted from MAC-T cells, bovine mammary tissues, and mouse mammary tissues using TRIzol Universal reagent (G3013, Servicebio, Wuhan, China). RNA concentration and purity were determined using a NanoDrop ND-1000 spectrophotometer (Thermo Fisher Scientific, Waltham, MA, USA), and samples with A_260_/A_280_ ratios of 1.8–2.0 were used for subsequent analyses. cDNA was synthesized using the Evo M-MLV Reverse Transcription Kit (AG11732, Accurate Biotechnology, Changsha, China), and quantitative real-time PCR was performed using SYBR Green Pro Taq HS Premix (AG11746, Accurate Biotechnology, Changsha, China). *β-Actin* was used as the reference gene, and relative gene expression was calculated using the 2^−ΔΔCt^ method. qPCR reactions were performed in technical triplicate. Primer sequences are listed in Supplementary Table S1.

### 2.6. Western Blot Analysis

Total proteins were extracted from MAC-T cells, bovine mammary tissues, and mouse mammary tissues using RIPA lysis buffer supplemented with 1% PMSF (Solarbio, Beijing, China). Protein concentrations were determined using a BCA Protein Assay Kit (Beyotime, Shanghai, China). Equal amounts of protein were separated by SDS-PAGE and transferred onto PVDF membranes. After blocking with 5% non-fat milk for 1 h, membranes were incubated with primary antibodies overnight at 4 °C, followed by HRP-conjugated secondary antibodies for 1 h at room temperature. Protein bands were visualized using enhanced chemiluminescence and quantified using ImageJ software (version 1.53t). β-Actin was used as the loading control. Information on the primary antibodies is provided in Supplementary Table S2.

### 2.7. Histopathological and Immunostaining Analyses

Mouse mammary tissues fixed in 4% paraformaldehyde were paraffin-embedded and sectioned at 4 μm. Hematoxylin and eosin (H&E) staining was performed to evaluate histopathological changes. For immunohistochemistry (IHC), sections were subjected to antigen retrieval, blocked with 5% BSA, incubated with primary antibodies overnight at 4 °C, and subsequently incubated with the corresponding secondary antibodies. Immunoreactivity was visualized using DAB followed by hematoxylin counterstaining.

For immunofluorescence (IF), MAC-T cells and mouse mammary tissue sections were processed using a multiplex fluorescence staining kit (AiFang Biological, Changsha, China) according to the manufacturer’s instructions. Samples were incubated with primary antibodies overnight at 4 °C and fluorescence-conjugated secondary antibodies for 1 h at 37 °C. Images were acquired using a microscope (Axiocam 208 color, Zeiss, Oberkochen, Germany) or an inverted fluorescence microscope (Revolve Omega, ApexBio, Suzhou, China). IHC-positive area, mean optical density, and fluorescence intensity were quantified using ImageJ software. For each animal, five randomly selected non-overlapping fields were analyzed, and the mean value was used as the animal-level measurement for statistical analysis (*n* = 6 mice per group). Histopathological scoring was independently performed by experienced experts blinded to group allocation using a standardized scoring system, with detailed criteria and representative images provided in Figure S3. IHC and IF image analyses were likewise performed blinded to group allocation.

### 2.8. Statistical Analysis

Data were analyzed using GraphPad Prism 9.0 (GraphPad Software, San Diego, CA, USA) and IBM SPSS Statistics 25.0. For bovine tissue analyses, each cow was considered one biological replicate (*n* = 4 per group); for mouse analyses, each animal was considered one biological replicate (*n* = 6 per group). MAC-T cell experiments were independently repeated three times. Normality and homogeneity of variance were assessed before parametric analysis. Comparisons between two groups were performed using an independent-samples *t*-test, whereas multiple-group comparisons were analyzed using one-way analysis of variance (ANOVA) followed by Tukey’s multiple-comparisons test. Data are presented as the mean ± standard deviation (SD), and *p* < 0.05 was considered statistically significant.

## 3. Results

### 3.1. Transcriptomic Analysis Reveals Inflammation- and Cytoskeleton-Related Molecular Alterations in Bovine Mastitis

**Figure 1:**
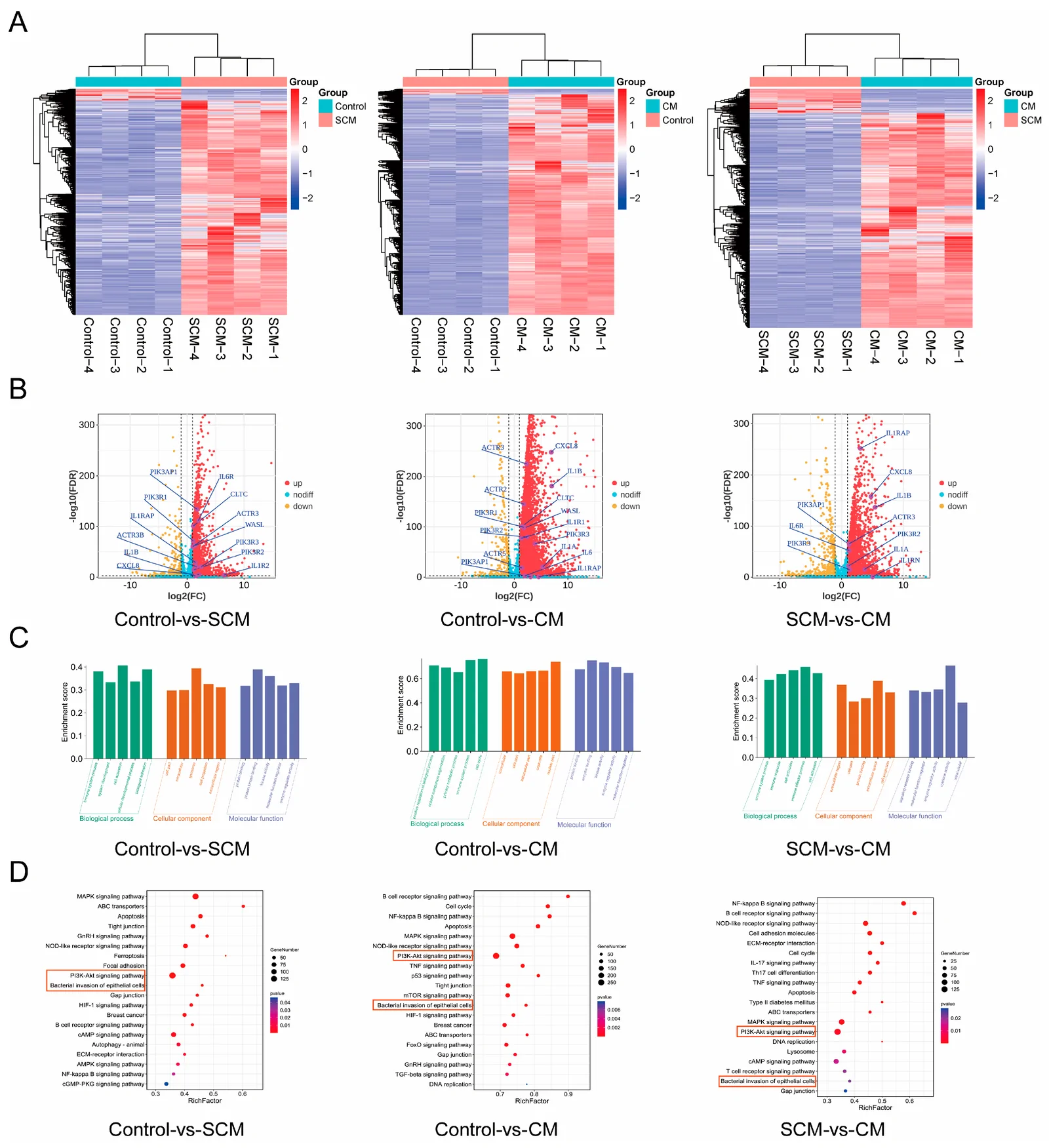
Transcriptomic analysis of bovine mammary tissues from the Control, SCM, and CM groups. (**A**) Hierarchical clustering heatmaps of differentially expressed genes (DEGs) among the Control, SCM, and CM groups. (**B**) Volcano plots of DEGs in the Control vs. SCM, Control vs. CM, and SCM vs. CM comparisons. Red and green dots represent upregulated and downregulated genes, respectively. (**C**) Gene Ontology (GO) enrichment analysis of DEGs, including biological process (BP), cellular component (CC), and molecular function (MF) categories. (**D**) Kyoto Encyclopedia of Genes and Genomes (KEGG) pathway enrichment analysis of DEGs in the three comparisons. DEGs were identified using the criteria |log2FC| ≥ 1 and FDR < 0.05. Control, healthy control; SCM, subclinical mastitis; CM, clinical mastitis; DEGs, differentially expressed genes. For all pairwise comparisons, positive log2FC values indicate higher expression in the second group, whereas negative values indicate lower expression.

Transcriptomic sequencing revealed distinct gene expression profiles among mammary tissues from the healthy control (Control, C), subclinical mastitis (SCM), and clinical mastitis (CM) groups. PCA showed clear separation among the Control, SCM, and CM groups with good within-group clustering (Figure S1A). Differential expression analysis identified 5923 DEGs in the C vs. SCM comparison, including 5621 genes upregulated in SCM relative to C and 302 genes downregulated in SCM relative to C; 12,431 DEGs in the C vs. CM comparison, including 12,119 genes upregulated in CM relative to C and 312 genes downregulated in CM relative to C; and 5762 DEGs in the SCM vs. CM comparison, including 5164 genes upregulated in CM relative to SCM and 598 genes downregulated in CM relative to SCM (Figure S1B).The largest number of DEGs was observed between the C and CM groups, indicating more extensive transcriptional alterations in clinical mastitis (Figure 1B). Hierarchical clustering analysis showed highly similar expression patterns among biological replicates within the same group, whereas the three groups were clearly distinguishable, further demonstrating marked transcriptomic differences among different stages of mastitis (Figure 1A). Volcano plot analysis revealed significant alterations in multiple inflammation- and cytoskeleton-related genes, including *IL1B*, *IL6*, *CXCL8*, *IL1R1*, *IL1RAP*, *PIK3R1*, *PIK3R2*, *PIK3R3*, *PIK3AP1*, *WASL*, *CLTC*, *ACTR2*, and *ACTR3* (Figure 1B). Gene Ontology (GO) enrichment analysis showed that the DEGs were broadly associated with biological process, cellular component, and molecular function categories (Figure 1C). Kyoto Encyclopedia of Genes and Genomes (KEGG) pathway analysis further demonstrated enrichment of DEGs in inflammation-, immunity-, and cytoskeleton-related pathways, including the PI3K-Akt signaling pathway, MAPK signaling pathway, NF-κB signaling pathway, NOD-like receptor signaling pathway, TNF signaling pathway, cell adhesion molecules, and bacterial invasion of epithelial cells (Figure 1D). Notably, the PI3K-Akt signaling pathway and bacterial invasion of epithelial cells pathway were prominently enriched across comparisons among different stages of mastitis (Figure 1D). Collectively, these findings indicate that the development and progression of bovine mastitis are accompanied by pronounced inflammatory responses and transcriptional alterations related to cytoskeletal regulation, and further suggest that PI3K- and N-WASP/ARP2/3-related molecules may participate in the pathological process of mastitis, providing a basis for subsequent validation of these candidate molecules.

### 3.2. Enhanced Inflammatory Responses and Increased Expression of PI3K/N-WASP/ARP2/3-Related Molecules in Bovine Mastitic Mammary Tissues

**Figure 2:**
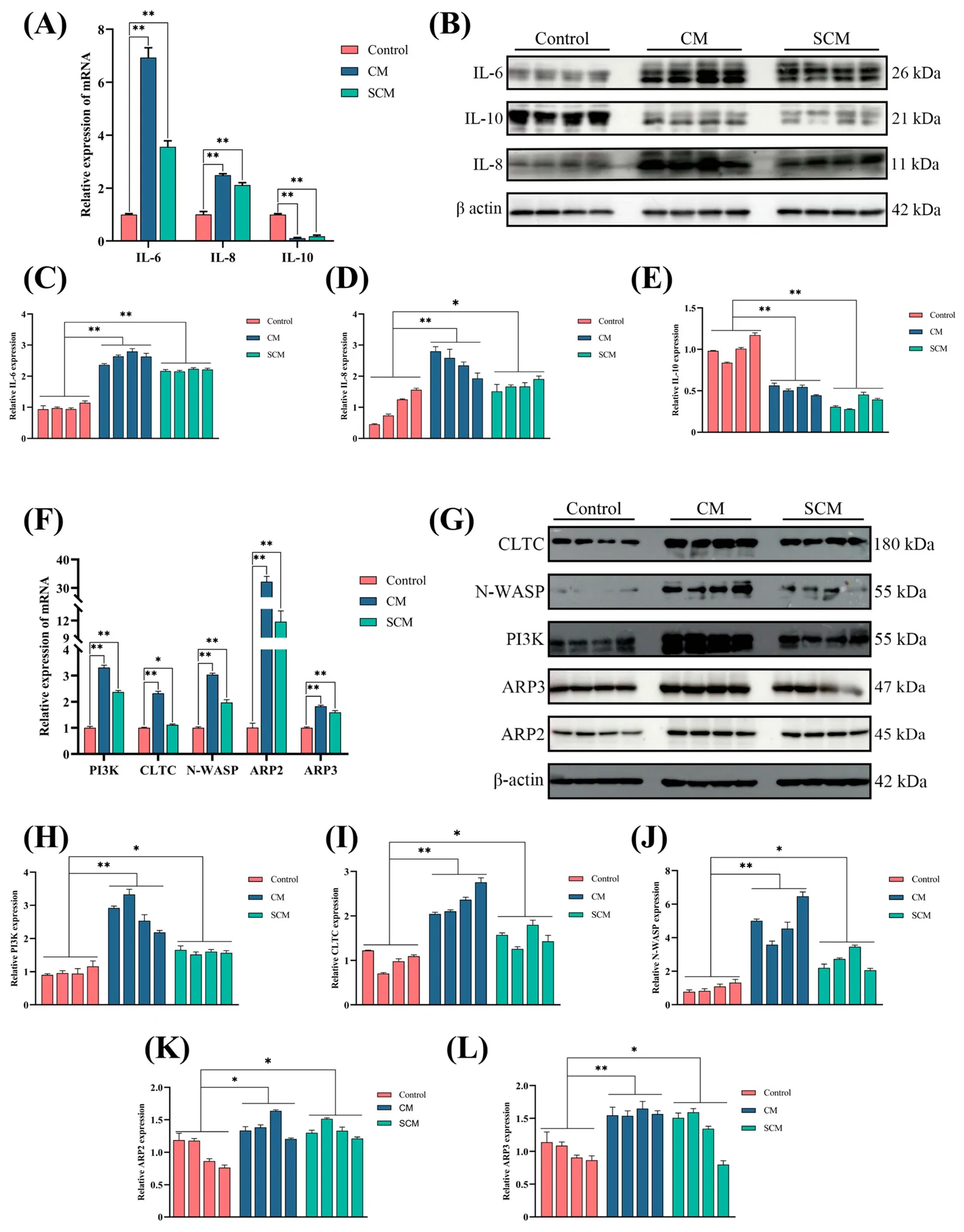
Expression of inflammatory cytokines and PI3K/N-WASP/ARP2/3-related molecules in bovine mastitic mammary tissues. (**A**) qPCR analysis of the mRNA expression levels of *IL-6*, *IL-8*, and *IL-10* in the Control, CM, and SCM groups. (**B**) Western blot analysis of IL-6, IL-10, and IL-8 protein expression. (**C**–**E**) Quantification of IL-6, IL-8, and IL-10 protein expression, respectively. (**F**) qPCR analysis of the mRNA expression levels of *PI3K*, *CLTC*, *N-WASP*, *ARP2*, and *ARP3*. (**G**) Western blot analysis of CLTC, N-WASP, PI3K, ARP3, and ARP2 protein expression. (**H**–**L**) Quantification of PI3K, CLTC, N-WASP, ARP2, and ARP3 protein expression, respectively. β-Actin was used as the loading control. Control, healthy control; SCM, subclinical mastitis; CM, clinical mastitis. * *p* < 0.05; ** *p* < 0.01. Data are presented as mean ± SD (*n* = 4 cows per group). Original Western blot images for Figure 2B,G are found in Supplementary Figures S4 and S5.

Based on the transcriptomic findings, qPCR and Western blot analyses were further performed to evaluate the expression of inflammatory cytokines and PI3K/N-WASP/ARP2/3-related molecules in mammary tissues from Control, SCM, and CM cows (Figure 2). Compared with the Control group, the mRNA and protein levels of the pro-inflammatory cytokines IL-6 and IL-8 were significantly increased in the SCM and CM groups, whereas the expression of the anti-inflammatory cytokine IL-10 was significantly decreased (Figure 2A–E). Meanwhile, the mRNA and protein expression levels of PI3K, N-WASP, CLTC, ARP2, and ARP3 were significantly elevated in mastitic mammary tissues (Figure 2F–L). These findings demonstrate that bovine mastitis is characterized by a marked imbalance in inflammatory cytokine expression together with increased expression of PI3K/N-WASP/ARP2/3-related molecules, further supporting the involvement of both inflammatory responses and cytoskeleton-associated regulatory alterations in mastitis development and progression.

### 3.3. CB Attenuates LPS-Induced Inflammatory Responses in MAC-T Cells and Mouse Mammary Tissues

**Figure 3:**
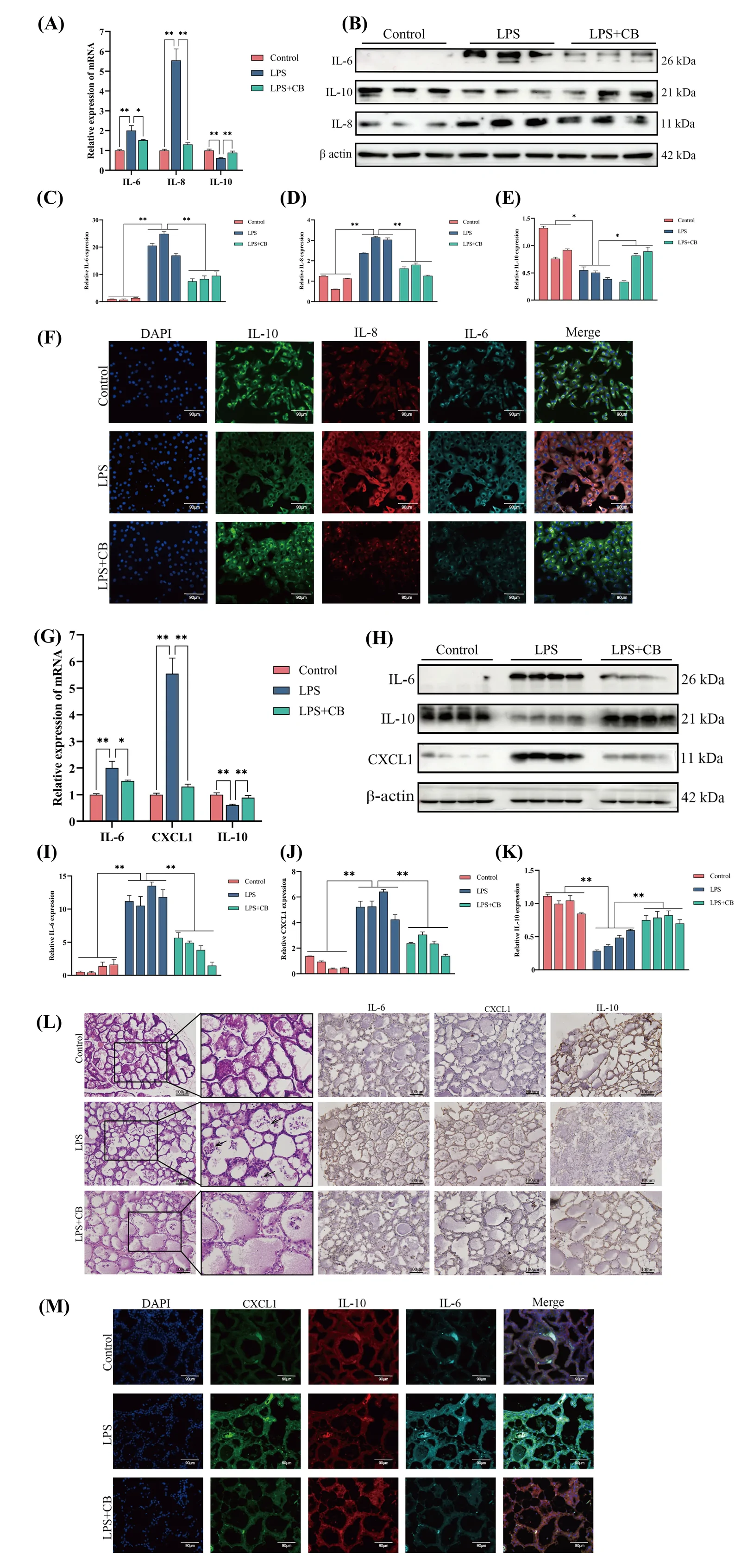
CB attenuates LPS-induced inflammatory responses in MAC-T cells and mouse mammary tissues. (**A**) qPCR analysis of the mRNA expression levels of *IL-6*, *IL-8*, and *IL-10* in MAC-T cells. (**B**) Western blot analysis of IL-6, IL-10, and IL-8 protein expression. (**C**–**E**) Quantification of IL-6, IL-8, and IL-10 protein expression, respectively. (**F**) Immunofluorescence analysis of IL-10, IL-8, and IL-6 expression in MAC-T cells. (**G**) qPCR analysis of the mRNA expression levels of *IL-6*, *CXCL1*, and *IL-10* in mouse mammary tissues. (**H**) Western blot analysis of IL-6, IL-10, and CXCL1 protein expression. (**I**–**K**) Quantification of IL-6, CXCL1, and IL-10 protein expression, respectively. (**L**) H&E staining of mouse mammary tissues and immunohistochemical analysis of IL-6, CXCL1, and IL-10 expression. (**M**) Immunofluorescence analysis of CXCL1, IL-10, and IL-6 expression in mouse mammary tissues. β-Actin was used as the loading control. Control, untreated control; LPS, lipopolysaccharide-treated group; LPS + CB, lipopolysaccharide plus cytochalasin B-treated group. * *p* < 0.05; ** *p* < 0.01. For panels A–F, data were obtained from three independent MAC-T cell experiments. For panels (**G**–**M**), *n* = 6 mice per group. Data are presented as mean ± SD. Original Western blots image for Figure 3B,H are found in Supplementary Figures S6 and S7.

To evaluate the anti-inflammatory effects of CB, a CCK-8 assay was first performed to determine a non-cytotoxic concentration in MAC-T cells. CB at concentrations up to 500 ng/mL had no significant effect on cell viability; therefore, 500 ng/mL was selected for subsequent experiments (Figure S2A). In the additional CB-alone control experiment, CB treatment alone did not significantly alter the protein expression of IL-1β, TNF-α, or IL-10 compared with the Control group. In contrast, CB treatment attenuated the LPS-induced increases in IL-1β and TNF-α and the decrease in IL-10 expression (Figure S2B). LPS stimulation significantly increased the expression of IL-6 and IL-8 while decreasing IL-10 expression in MAC-T cells. These alterations were markedly reversed by CB treatment, with consistent trends observed by qPCR, Western blotting, and immunofluorescence analyses (Figure 3A–F). In the LPS-induced mouse mastitis model, LPS caused substantial mammary tissue damage, epithelial cell shedding, and prominent inflammatory cell infiltration (Figure 3L), accompanied by increased IL-6 and CXCL1 expression and decreased IL-10 expression (Figure 3G–K,M). CB treatment markedly alleviated the histopathological damage and inflammatory cell infiltration (Figure 3L), reduced IL-6 and CXCL1 expression, and increased IL-10 expression (Figure 3G–M). Collectively, these results demonstrate that CB consistently attenuates LPS-induced mammary inflammatory responses and tissue injury both in vitro and in vivo.

### 3.4. CB Reduces the Expression of PI3K/N-WASP/ARP2/3-Related Molecules in LPS-Stimulated MAC-T Cells and Mouse Mammary Tissues

**Figure 4:**
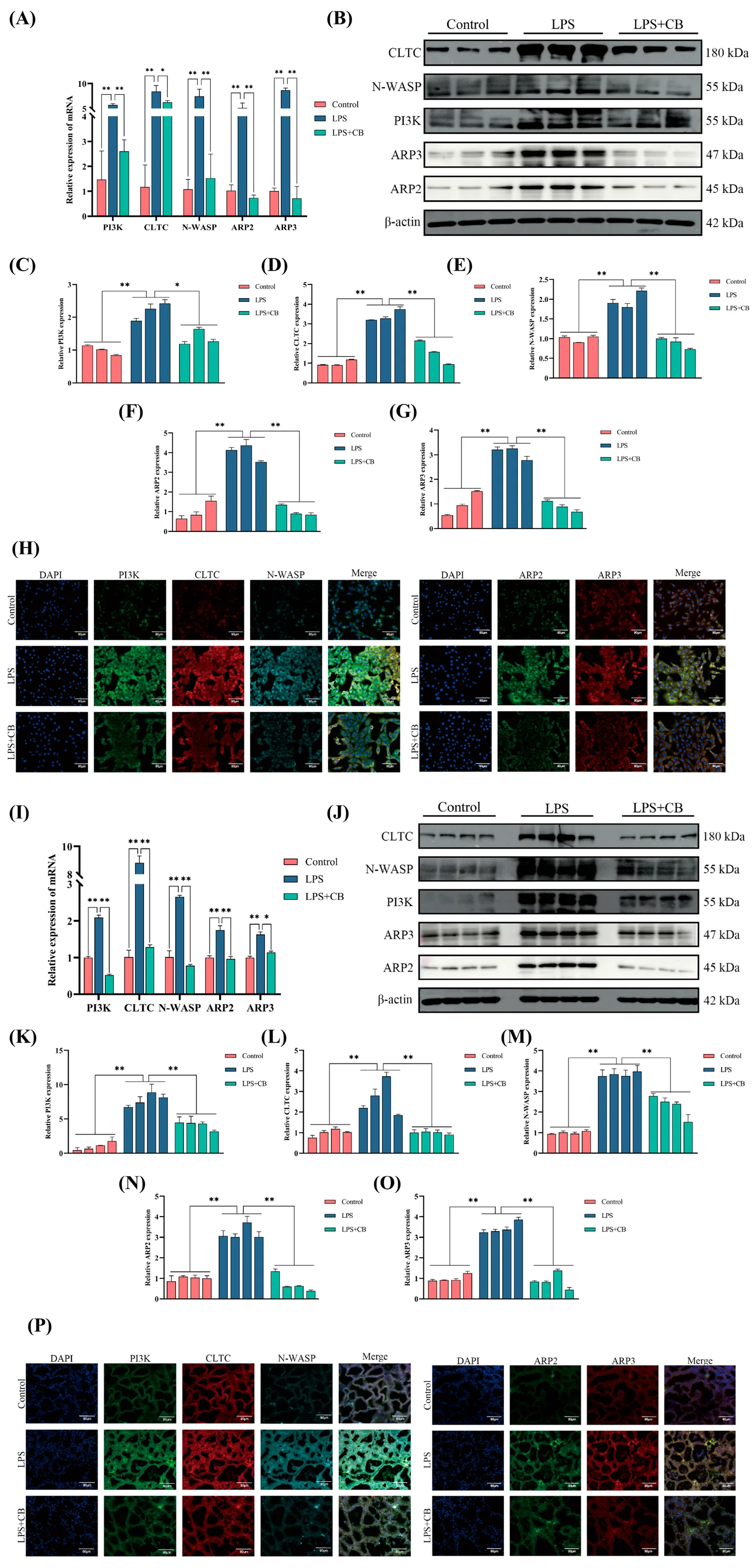
CB reduces the expression of PI3K/N-WASP/ARP2/3-related molecules in LPS-stimulated MAC-T cells and mouse mammary tissues. (**A**) qPCR analysis of the mRNA expression levels of *PI3K*, *CLTC*, *N-WASP*, *ARP2*, and *ARP3* in MAC-T cells. (**B**) Western blot analysis of the corresponding proteins. (**C**–**G**) Quantification of PI3K, CLTC, N-WASP, ARP2, and ARP3 protein expression, respectively. (**H**) Immunofluorescence analysis of PI3K, CLTC, N-WASP, ARP2, and ARP3 expression in MAC-T cells. (**I**) qPCR analysis of the mRNA expression levels of PI3K, CLTC, N-WASP, ARP2, and ARP3 in mouse mammary tissues. (**J**) Western blot analysis of the corresponding proteins. (**K**–**O**) Quantification of PI3K, CLTC, N-WASP, ARP2, and ARP3 protein expression, respectively. (**P**) Immunofluorescence analysis of PI3K, CLTC, N-WASP, ARP2, and ARP3 expression in mouse mammary tissues. β-Actin was used as the loading control. Control, untreated control; LPS, lipopolysaccharide-treated group; LPS + CB, lipopolysaccharide plus cytochalasin B-treated group. * *p* < 0.05; ** *p* < 0.01. For panels (**A**–**H**), data were obtained from three independent MAC-T cell experiments. For panels (**I**–**P**), *n* = 6 mice per group. Data are presented as mean ± SD. Original Western blot images for Figure 4B,J are found in Supplementary Figures S8 and S9.

To further investigate the relationship between the anti-inflammatory effects of CB and alterations in PI3K/N-WASP/ARP2/3-related molecules, the expression of PI3K, N-WASP, CLTC, ARP2, and ARP3 was examined in both MAC-T cells and the mouse mastitis model (Figure 4). LPS treatment significantly increased the mRNA expression of these molecules in MAC-T cells, whereas CB markedly attenuated their LPS-induced upregulation (Figure 4A). Consistent changes at the protein level were further confirmed by Western blotting and quantitative analysis (Figure 4B–G), as well as by immunofluorescence staining (Figure 4H). Similarly, LPS markedly increased the mRNA expression of PI3K, N-WASP, CLTC, ARP2, and ARP3 in mouse mammary tissues, whereas CB treatment significantly reversed these changes (Figure 4I). Western blotting and quantitative analyses showed corresponding changes in protein expression (Figure 4J–O), which were further supported by immunofluorescence staining (Figure 4P). Taken together, the in vitro and in vivo findings indicate that CB attenuates LPS-induced inflammatory responses accompanied by reduced expression of PI3K/N-WASP/ARP2/3-related molecules, suggesting an association between these molecular alterations and the anti-inflammatory effects of CB.

## 4. Discussion

Bovine mastitis is a major disease affecting dairy cow health, milk quality, and the economic efficiency of dairy production, and its development is accompanied by complex inflammatory responses and disruption of mammary tissue homeostasis [25,26]. In the present study, transcriptomic sequencing was first used to compare gene expression profiles in mammary tissues from healthy cows and cows with subclinical or clinical mastitis. Distinct transcriptomic differences were observed among the three mammary health states, with the greatest number of differentially expressed genes detected between the clinical mastitis and healthy groups, indicating more extensive transcriptional remodeling as disease severity increased. Further validation in bovine mammary tissues showed significantly increased expression of IL-6 and IL-8 and decreased expression of IL-10 in the SCM and CM groups, together with marked increases in the mRNA and protein levels of PI3K, N-WASP, CLTC, ARP2, and ARP3. These findings indicate that mastitis-associated inflammatory responses are accompanied by alterations in molecules related to cytoskeletal regulation and membrane trafficking, suggesting a close association between these processes during mastitis progression.

Aberrant expression of inflammatory mediators is a hallmark of mastitis. Previous studies have shown that LPS induces robust expression of pro-inflammatory cytokines, including IL-1β, IL-6, IL-8, and TNF-α, in bovine mammary epithelial cells, thereby promoting local inflammatory injury [27,28,29,30,31]. Consistently, LPS stimulation in the present study markedly increased IL-6 and IL-8 expression in MAC-T cells, whereas CB treatment reduced these pro-inflammatory mediators and increased IL-10 expression. These findings are consistent with our previous study showing that CB suppressed LPS-induced increases in IL-1β and TNF-α expression in mammary epithelial cells [24]. In the mouse mastitis model, LPS increased IL-6 and CXCL1 expression, decreased IL-10 expression, and caused alveolar structural disruption, mammary epithelial cell shedding, and inflammatory cell infiltration; these changes were substantially alleviated by CB treatment. Previous studies have reported that allicin, JMJD3 inhibitors, *Lactobacillus plantarum*, and active compounds derived from *Forsythia* can attenuate LPS-induced mammary inflammation by suppressing inflammatory mediators or modulating related signaling pathways [32,33,34,35,36]. In agreement with these findings, our in vitro and in vivo results demonstrate that CB effectively alleviates LPS-induced mammary inflammation and tissue injury, further supporting its potential biological value in the regulation of mastitis-associated inflammation.

Cytoskeletal dynamics are increasingly recognized as being closely linked not only to cell morphology and motility but also to pathogen recognition, membrane trafficking, cell adhesion, and inflammatory signal transduction. PI3K participates in diverse processes, including cell survival, metabolism, immune inflammation, and cytoskeletal regulation [37]. N-WASP is a key regulator of actin polymerization that promotes ARP2/3 complex-mediated formation of branched actin networks and cytoskeletal remodeling [38,39]. The WASP family and ARP2/3 complex are also involved in autophagy, apoptosis, and other cellular stress responses [40], whereas FAK/N-WASP/ARP2/3-related regulation has been shown to contribute to cytoskeletal remodeling and cell migration in multiple cell types [41,42]. In the present study, PI3K, N-WASP, ARP2, and ARP3 were markedly increased in mammary tissues from SCM and CM cows and showed similar expression patterns in LPS-stimulated MAC-T cells and mouse mammary tissues, indicating a close association between mastitis-associated inflammatory responses and alterations in PI3K- and N-WASP/ARP2/3-related molecules. Meanwhile, the parallel changes in CLTC suggest that molecules associated with clathrin-mediated endocytosis and membrane trafficking are also altered during inflammatory stimulation.

**Figure 5:**
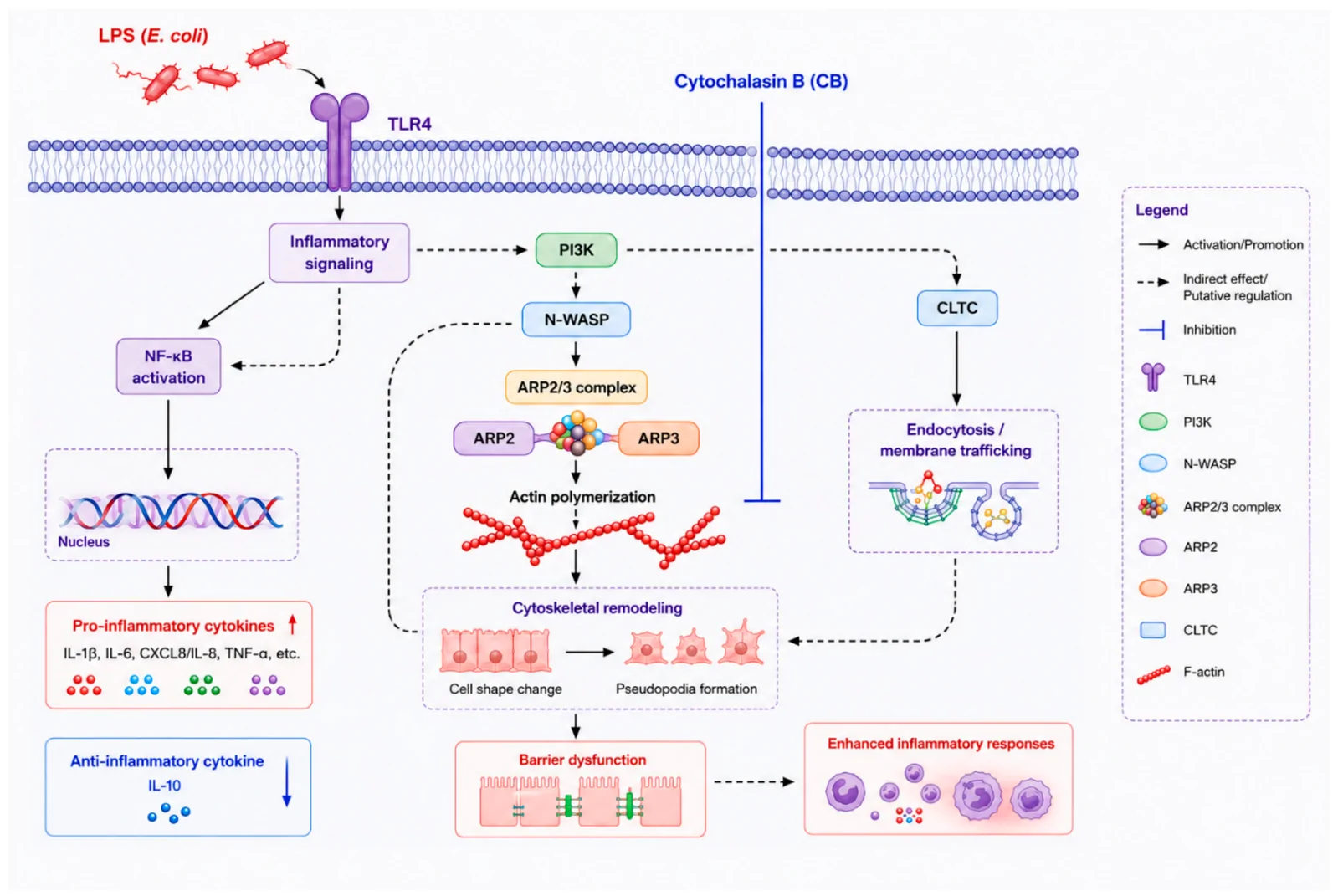
Schematic summary of molecular changes associated with cytochalasin B (CB) treatment in LPS-induced mammary inflammation. In the present study, LPS-induced mammary inflammation was accompanied by altered inflammatory cytokine expression and increased expression of PI3K, N-WASP, CLTC, ARP2, and ARP3. Previous studies have established LPS/TLR4-associated inflammatory signaling [32], the role of N-WASP/ARP2/3 in actin regulation [43], and the involvement of CLTC in clathrin-mediated endocytosis and membrane trafficking [46]. CB inhibits actin polymerization [18] and, based on the present experimental findings, may attenuate these inflammatory and cytoskeleton-related alterations. Solid arrows indicate activation or promotion supported by previous studies, dashed arrows indicate potential associations proposed based on the present experimental findings and existing literature, and blunt-ended lines indicate inhibition.

In the present study, CB attenuated LPS-induced inflammatory responses and markedly reduced the aberrant upregulation of PI3K, N-WASP, CLTC, ARP2, and ARP3 in both MAC-T cells and mouse mammary tissues. Given the pivotal role of N-WASP/ARP2/3 in branched actin remodeling [43,44], together with the involvement of PI3K in inflammatory and cytoskeletal regulation and CLTC in clathrin-mediated membrane trafficking [45,46], these findings suggest that the protective effects of CB may be associated with PI3K/N-WASP/ARP2/3-related cytoskeletal regulation and CLTC-associated membrane trafficking. A schematic representation of the proposed molecular mechanism is shown in Figure 5. However, as the present study primarily characterized changes at the transcriptional and total protein levels, further functional studies and direct assessment of cytoskeletal dynamics are warranted to clarify the specific roles of these molecules in the protective effects of CB. In addition, the LPS-induced mastitis model represents sterile inflammation rather than active bacterial infection and therefore does not address bacterial clearance or pathogen survival. It should also be noted that, because a vehicle-matched control was not included in the mouse experiment, the potential contribution of the approximately 2% DMSO-containing vehicle to the observed in vivo effects cannot be entirely excluded. The potential effects of CB on host defense and its safety also require further investigation.

## 5. Conclusions

This study demonstrates that bovine mastitis is associated with enhanced inflammatory responses and abnormal expression of PI3K/N-WASP/ARP2/3-related molecules. CB effectively alleviated LPS-induced mammary inflammation and tissue injury in both MAC-T cells and mice, while reducing the aberrant expression of PI3K, N-WASP, CLTC, ARP2, and ARP3. Collectively, these findings suggest that the anti-inflammatory effects of CB are closely associated with alterations in PI3K/N-WASP/ARP2/3-related molecules, providing further evidence for a link between cytoskeletal regulation and inflammatory responses in mastitis.

## Data Availability

The raw RNA-sequencing data generated in this study are available in the National Center for Biotechnology Information (NCBI) Sequence Read Archive (SRA) under BioProject accession number PRJNA1314066. All other data supporting the findings of this study are available within the article and its Supplementary Materials (dongwt@gsau.edu.cn (W.D.)).

## Supplementary Materials

The following supporting information can be downloaded at: https://www.mdpi.com/article/10.3390/biom16101467/s1, Figure S1: Overview of RNA-seq sample distribution and differentially expressed genes among the Control, SCM, and CM groups. Figure S2: Effects of CB alone and CB treatment on cell viability and inflammatory responses in MAC-T cells. Figure S3: Representative H&E-stained images and standardized histopathological scoring criteria for mouse mammary tissue injury. Figures S4–S10: Original Western blot images corresponding to the Western blot analyses in the manuscript and Supplementary Materials. Table S1: Primer information. Table S2: Antibody information.
