# Supplementary material for: Cytochalasin B Alleviates LPS-Induced Mastitis and Modulates the Expression of PI3K/N-WASP/ARP2/3-Related Molecules
Source: Biomolecules. 2026 Oct 9;16(10):1467. doi: 10.3390/biom16101467 (PMC13702464; doi:10.3390/biom16101467)

β actin

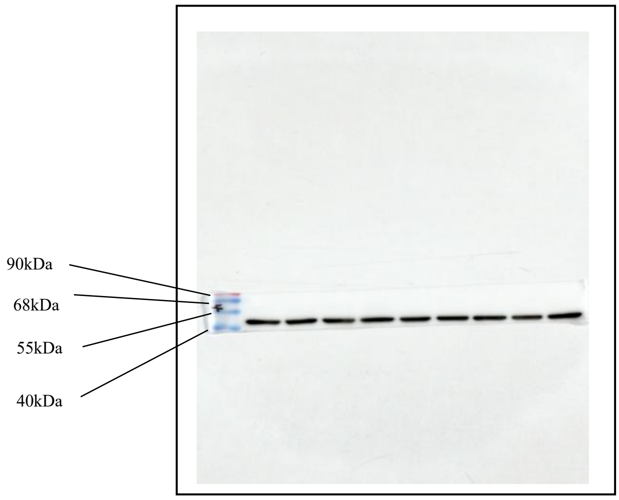

IL-6

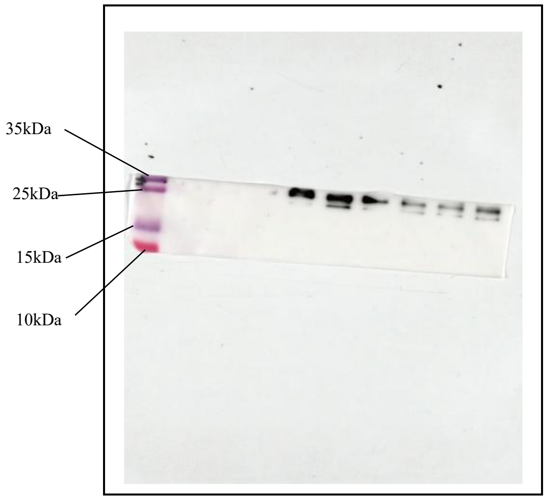

IL-8

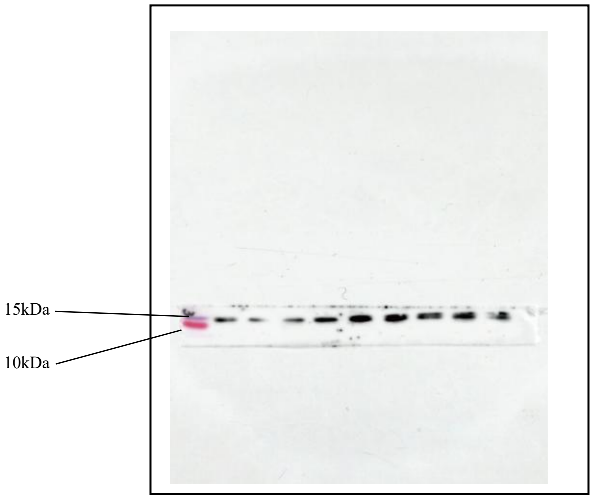

IL-10

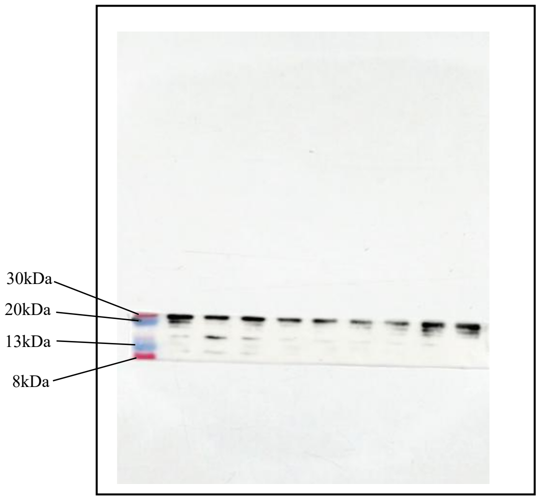

Supplement: Supplementary file 1 [file biomolecules-16-01467-s001.zip › Figure S6.pdf]
