# Supplementary material for: Cytochalasin B Alleviates LPS-Induced Mastitis and Modulates the Expression of PI3K/N-WASP/ARP2/3-Related Molecules
Source: Biomolecules. 2026 Oct 9;16(10):1467. doi: 10.3390/biom16101467 (PMC13702464; doi:10.3390/biom16101467)
Supplement: Supplementary file 1 [file biomolecules-16-01467-s001.zip › Table S1.pdf]

Table 1 The information of all primers

Species	Gene	GenBank NO.	Sequence (5′-3′)	Length（bp）
Mouse	*IL-6*	NM_173923.2	TCCTGAAGCAAAAGATCGCA	221
			CTGACCAGAGGAGGGAATGC	
	CXCL1	NM_008361.4	GCCACCTTTTGACAGTGATGAG	135
			ATGTGCTGCTGCGAGATTTG	
	IL-10	XM_036162094.1	CTTACTGACTGGCATGAGGATCA	101
			GCAGCTCTAGGAGCATGTGG	
	CLTC	XM_006533983.3	TTGCCAACATTGCCATCAGC	145
			TTGCAGCGTTCAGCAAACTC	
	PI3K	NM_001024955.2	CACCACGGTTTGGACTATGGA	138
			GCTACAGTAGTGGGCTTGGG	
	N-WASP	NM_028459.2	AATAGTCGGCCCGTGTCCT	99
			GGTGGACTCTTGGCCATC	
	ARP2	NM_146243.2	CCCAGCTTTGGTTGGAAGAC	74
			CCATAAGGTCCTTGATTTCAATGTT	
	ARP3	NM_001205385.1	GACAAGTAGGAGAGCGGACG	195
			AGGACTGCTCAGGAGGGATT	
	β-actin	NM_007393.5	GGCTGTATTCCCCTCCATCG	154
			CCAGTTGGTAACAATGCCATGT	
Bovine	*IL-6*	NM_031168.2	GCTGAATCTTCCAAAAATGGAGG	200
			GCTTCAGGATCTGGATCAGTG	
	IL-8	NM_173925.2	AAGCTGGCTGTTGCTCTCTT	127
			GGTGGAAAGGTGTGGAATGT	
	IL-10	NM_174088.1	TTGCCAAGCCTTGTCGGAAA	114
			TTCAGCTTCTCCCCCAGTGA	
	CLTC	NM_174023.2	TGCAATGCAGTATGCCTCTGA	237
			GCATCTAATTTATCCACCTTTGTCA	
	PI3K	XM_024993013.2	CATCTCCACAGCTACGCCAT	231
			AGCCATTCATTCCACCTGGG	
	N-WASP	XM_010804401.3	TTGGACATGTGGGTTGGGAT	193
			GCTTGCCTTCGCAGTTCATT	
	ARP2	NM_001102213.1	GCCGGGAGGGATATTACCAG	213
			CCCGACTTTGATAATGCGGC	
	ARP3	NM_174226.2	TTGCTGTACAGGCCGTTCTT	177
			TATCTCGTCCTGCGATGGGA	
	β-actin	AY141970.1	CAACCGTGAGAAGATGACCCA	293
			TGTCACGGACGATTTCCGCTC	
