# Supplementary material for: Cytochalasin B Alleviates LPS-Induced Mastitis and Modulates the Expression of PI3K/N-WASP/ARP2/3-Related Molecules
Source: Biomolecules. 2026 Oct 9;16(10):1467. doi: 10.3390/biom16101467 (PMC13702464; doi:10.3390/biom16101467)
Supplement: Supplementary file 1 [file biomolecules-16-01467-s001.zip › Table S2 .pdf]

Table 2 The information of all antibodies

Name	Manufacturer	Cat.NO.	Host species	Samples used in this study	Working dilution
IL-6	Affinity	DF6087	Rabbit	Bovine, MAC-T, Mouse	WB 1:1000; IHC 1:200; IF 1:200
IL-8	Affinity	DF6998	Rabbit	Bovine, MAC-T	WB 1:1000; IF 1:200
CXCL1	Abcam	AB269939	Rabbit	Mouse	
IL-10	Affinity	DF6894	Rabbit	Bovine, MAC-T, Mouse	WB 1:1000; IHC 1:100; IF 1:200
CLTC	Abmart	T56862	Rabbit	Bovine, MAC-T, Mouse	WB 1:1000; IHC 1:100; IF 1:200
PI3K	Abmart	T40115	Rabbit	Bovine, MAC-T, Mouse	WB 1:1000; IF 1:200
N-WASP	Abmart	TN25205	Rabbit	Bovine, MAC-T, Mouse	WB 1:1000; IF 1:200
ARP2	Bioss	bsm-62309R	Rabbit	Bovine, MAC-T, Mouse	WB 1:8000; IF 1:200
ARP3	Bioss	bsm-54337R	Rabbit	Bovine, MAC-T, Mouse	WB 1:500; IF 1:200
β-actin	Proteintech	66009-1-IG	Rabbit	Bovine, MAC-T, Mouse	WB 1:10,000
